# Comprehensive analysis of protective effects of *Loranthus tanakae* Franch. and Sav. on ovalbumin-induced atopic dermatitis in mice and TNF-*α*/INF-*γ*-stimulated HaCaT cells

**DOI:** 10.1080/19768354.2025.2498928

**Published:** 2025-05-14

**Authors:** Yea-Gin Yang, Woong-Il Kim, A. Yeong Lee, So-Won Pak, Sin-Hyang Park, Young-Kwon Cho, Joong-Sun Kim, Jong-Choon Kim, Sung-Hwan Kim, In-Sik Shin

**Affiliations:** a College of Veterinary Medicine and BK21 FOUR Program, Chonnam National University, Buk-gu, Gwangju, Republic of Korea; b KM Data Division, Korea Institute of Oriental Medicine, Daejeon, Republic of Korea; c College of Health Sciences, Cheongju University, Sangdang-gu, Cheongju-si, Republic of Korea; d Jeonbuk Branch, Korea Institute of Toxicology (KIT), Jeongeup-si Jeonbuk, Republic of Korea

**Keywords:** Atopic dermatitis, *Loranthus tanakae* Franch. & Sav*.*, ovalbumin, JAK2, STAT1

## Abstract

*Loranthus tanakae* Franch. and Sav. is a traditional herbal remedy with anti-inflammatory and antioxidative properties, used to treat joint and respiratory inflammation. In this study, we investigated the therapeutic effects of *L. tanakae* ethanol extract (LTE) on atopic dermatitis (AD). An ovalbumin (OVA)-induced AD animal model and a human keratinocyte cell line, HaCaT, were used to assess LTE treatment effects on AD. An *in vitro* experiment showed that LTE treatment significantly decreased the production of regulated upon activation, normal T cell expressed and secreted (RANTES) cytokines and macrophage-derived chemokines (MDC) in tumor necrosis factor-alpha (TNF-*α*)/interferon-gamma (IFN-*γ*) (TNF-*α*/IFN-*γ*)-stimulated HaCaT cells in a concentration-dependent manner. In addition, treatment with LTE markedly reduced the translocation of signal transducer and activator transcription 1 (STAT1) protein to the nucleus and the phosphorylation of Janus kinase 2 (JAK2) in TNF-*α*/IFN-*γ*-stimulated HaCaT cells. In the *in vivo* experiment, administration of LTE significantly decreased the levels of immunoglobulin E (IgE) and interleukin-13 (IL-13) of OVA-induced AD mice, which was supported by histological evidence. Moreover, LTE treatment markedly reduced inflammatory cell infiltration and edema in the OVA-induced AD mice’s damaged lesions. In addition, applying LTE notably inhibited the phosphorylation of JAK2 and STAT1 in the OVA-induced AD mice, supported by *in vitro* results. In conclusion, LTE effectively alleviated the AD-induced skin inflammation in the OVA-induced AD animal model and TNF-*α*/IFN-*γ*-stimulated HaCaT cells; this was related to the suppression of JAK2 and STAT1 phosphorylation. These findings suggest that LTE has potential as a therapeutic agent for AD management.

## Introduction

Atopic dermatitis (AD) is the most prevalent chronic skin disorder, affecting a considerable portion of the population across all age groups (Woo and Kim 2023). In particular, AD affects approximately 25% of children, making it the most common dermatological condition according to an epidemiological study (Kim and Leung 2018). The development of AD is closely associated with various immunological disorders (Ahn et al. 2024). In general, the prevalence of food allergies, asthma, allergic rhinitis, and other immune-mediated diseases in patients with AD is higher than in healthy people (Weidinger and Novak 2016). Atopic dermatitis is characterized by the development of eczematous lesions, which result from the interplay between impaired skin barrier function and exposure to allergens (Tsoi et al. 2020; Maintz et al. 2022; Nakashima et al. 2022). This pathophysiological alteration triggers the release of epithelial cytokines, including thymic stromal lymphopoietin (TSLP) and interleukin-13 (IL-13) (Ebina-Shibuya and Leonard 2023). These cytokines are important in immune responses and lead to the polarization and proliferation of T lymphocytes, primarily promoting their differentiation into T helper 2 (Th2) cells, which is a hallmark of allergic responses (Boguniewicz and Leung 2011; Zhu et al. 2011; Salazar-Espinosa 2014).

Despite considerable progress in dermatological treatments, a cure that fully treats the symptoms of AD remains unavailable. Currently, a recommended strategy to treat AD is using oral or topical corticosteroids, which helps reduce the abnormal immune responses typical of the condition (Roekevisch et al. 2014; Ross 2023). However, their long-term use is limited by their significant adverse effects, such as thinning of the epidermis and dermis, visible blood vessels (telangiectasia), immunosuppression, and changes in skin pigmentation (Abraham and Roga 2014; Waljee et al. 2017; Lugović-Mihić et al. 2023; Jang et al. 2024). Therefore, there is a critical need in dermatology to develop new AD treatments with improved safety profiles. Accordingly, developing natural product-based therapeutics that are effective in treating AD and have fewer adverse effects is very attractive.

*Loranthus tanakae* (*L. tanakae*) French. and Sav., a hemiparasitic plant native to East Asian countries including China, Japan, and Korea, has attracted considerable interest in ethnopharmacological studies because of its long history in traditional medicine (Park et al. 2022). Historically, *L. tanakae* has been used to treat many medical conditions, including tumors, inflammatory joint diseases, and various respiratory disorders (Zhou et al. 2021). Recent scientific studies have revealed a variety of activities, including antimicrobial, anticancer and antioxidant effects of biologically active small molecules obtained from nature, which support the use of *L.tanakae* in traditional medicine and indicate a diverse pharmacological profile (Qiu and Lin 1988; Kim et al. 2022). Although *L. tanakae* has shown promising therapeutic potential in various medical fields, there is a notable lack of research on its effectiveness in treating AD. Therefore, to fill this knowledge gap, our study investigated and evaluated the therapeutic effects of *L. tanakae* on AD using tumor necrosis factor-alpha (TNF-*α*) and interferon-gamma (IFN-*γ*) (TNF-*α*/IFN-*γ*)-stimulated human epidermal keratinocytes (HaCaT) and ovalbumin (OVA)-induced mouse model of AD.

## Materials and methods

### Preparation of L. tanakae for analysis

The aerial parts of *L. tanakae* were obtained from a herbalist in Jeongseon, Gangwon-do, Republic of Korea. The plant specimen was assigned voucher number 2-16-0335 and underwent verification at the Korea Institute of Oriental Medicine (KIOM). This authentication process was carried out by Dr. Sungyu Yang in collaboration with Dr. Byeong Cheol Moon. After authentication, the plant material was air-dried, ground into a fine powder using a blender (Hanil Industrial Machine, Seoul, Republic of Korea), and passed through a 600 μm mesh sieve.

### Process of extraction LTE from L. tanakae

The *L. tanakae* (1.032 kg) underwent reflux extraction twice, each time using 6 L of 70% ethanol for 2 h. The extracted solution was then passed through a chromatography filter sheet to remove impurities. Following filtration, the solvent was removed using a vacuum evaporation process. This procedure yielded 126.42 g of *L. tanakae* 70% ethanol extract (LTE), representing a 12.25% extraction efficiency by weight. The resulting LTE was preserved at a temperature of −20 °C for subsequent use.

### Analysis of LTE’s active ingredients

High-performance liquid chromatography (HPLC)-ultraviolet (UV) (HPLC-UV) analysis was performed on the LTE using an Agilent 1260 Series HPLC (Agilent Technologies Inc., Santa Clara, CA, USA). Chromatography was carried out on a C18 column (2.1 × 150 mm, 3.5 μm; Agilent) at below 40℃. The index standard chemicals, quercitrin and afzelin, were Sigma-Aldrich (Merck KGaA, Darmstadt, Germany; > 95% purity). The mobile phase consisted of 0.1% formic acid (Solvent A) and acetonitrile (Solvent B) in the gradient elution as follows: 0−1 min 3% B; 1−2 min 3%−15% B; 2−13 min 15%−50% B; 13−20 min 50%−100% B; 20−23 min 100% B; and 23−23.5 min 100%−3% at flow rate of 0.25 mL/min. The injection volume was set to 5 μL and the detection wavelength to 254 or 280 nm.

### Cell viability analysis

The HaCaT cells were maintained in a growth medium consisting of Roswell Park Memorial Institute (RPMI)−1640 enriched with 10% heat-inactivated fetal bovine serum (WELGENE Inc., Gyeongsan, Republic of Korea) and appropriate antibiotics. The cells were incubated at 37°C in a humidified atmosphere containing 5% CO2. An EZ-Cytox kit (DoGenBio Co., Ltd., Seoul, Republic of Korea) was used to evaluate cell viability following the manufacturer's protocol.

### Evaluation of inflammatory cytokines in HaCaT cells

The HaCaT cells were distributed into 12-well plates with each well containing 2 × 10^5^ cells. Following a 24-hour incubation period, the cells were exposed to varying concentrations of LTE (12.5, 25, 50, and 100 μg/mL). The cells were then incubated for an additional 2 h before being stimulated with TNF-*α*/IFN-*γ* (10 ng/mL) (PeproTech, Thermo Fisher Scientific Inc., Waltham, MA, USA) for 12 h. After the treatment period, the cell culture supernatant was collected. The concentrations of regulated upon activation, normal T cell expressed and secreted (RANTES) chemokine, macrophage-derived chemokine (MDC), and interleukin-8 (IL-8) (R&D Systems, Inc., Minneapolis, MN, USA) in the supernatant were quantified using enzyme-linked immunosorbent assay (ELISA) kits according to the manufacturer's instructions.

### Immunofluorescence analysis in HaCaT cells

The HaCaT cells were seeded on 6-well plates at a density of 2 × 10^5^ cells per well and incubated for 24 h. Then, the cells were treated with LTE at a concentration of 100 μg/mL. After 2 h of incubation, the cells were exposed to 10 ng/mL of TNF-*α*/IFN-*γ* for 12 h. Following this, the cells were fixed with 4% paraformaldehyde and permeabilized, then blocked with 5% bovine serum albumin (Sigma-Aldrich, Merck KGaA, Darmstadt, Germany). Subsequently, the same experiment was conducted using phospho-Janus kinase 2 (P-JAK2) and phospho-signal transducer and activator transcription 1 (P-STAT1) (Cell Signaling Technology, Inc., Danvers, MA, USA) antibodies; the results were observed with a confocal laser scanning microscope (ZEISS Microscopy, Jena, Germany).

### Animals

Female Balb/c mice (6 weeks old) were obtained from Samtako BIO KOREA Co., Ltd., Osan, Republic of Korea, and housed under standard conditions with unrestricted access to food and water. This study’s animal experiments were performed in compliance with the guidelines of the Ethics Committee for Animal Research at Chonnam National University. The study protocol received official authorization (CNU IACUC-YB-R-2023-10) before any *in vivo* experiments commenced.

### Ovalbumin-induced AD

**Figure 1.**
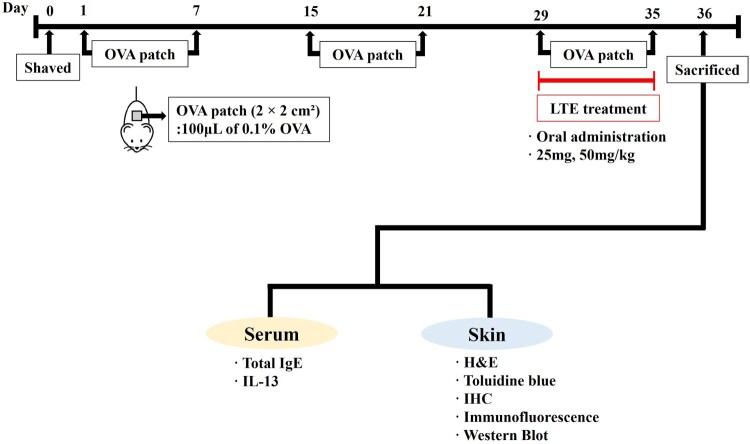
Scheme of atopic dermatitis induction and LTE treatment. H&E, hematoxylin and eosin stain; IgE, immunoglobulin E; IL-13, interleukin-13; IHC, immunohistochemistry staining; OVA, ovalbumin.

After inducing anesthesia by the inhalation of isoflurane, the dorsal skin of the mice was shaved. A gauze patch measuring 2 × 2 cm², soaked in either 100 μL of 0.1% OVA (Sigma-Aldrich, Merck KGaA, Darmstadt, Germany) or 0.9% of saline, was administered to the dorsal skin of each mouse and fixed in place using Tegaderm (3M Company, Saint Paul, MN, USA). The OVA or saline patches were left in place for 1 week and then removed for 1 week before being reapplied (Figure 1). The complete experiment consisted of three 1-week periods of OVA exposure, as shown in Figure 1.

### Measurement of serum inflammatory mediators

The serum levels of total IgE (BioLegend, San Diego, CA, USA) and IL-13 were quantified with commercial ELISA kits according to the manufacturer's protocols.

### Histopathology of mice skin tissue

The mice’s formalin-fixed skin tissue was subjected to standard hematoxylin and eosin staining procedures to evaluate its inflammatory responses. In addition, toluidine blue staining was used to assess the infiltration of mast cells into the skin’s damaged lesions. Immunohistochemistry was performed to evaluate the expression of P-JAK2 and P-STAT1 (diluted 1:100) using the method reported by Kim et al. (2023).

### Immunofluorescence to analyze P-JAK2 and P-STAT1 in mice skin tissue

The paraffin-embedded skin tissue sections on the slides were subjected to deparaffinization. Following this, the sections were incubated overnight at 4 °C with primary antibodies against P-STAT1 (diluted 1:100). Subsequently, the sections were reacted with a secondary antibody conjugated with fluorescein isothiocyanate (diluted 1:1,000; Cell Signaling Technologies Inc., Danvers, MA, USA). Subsequently, 4′,6-diamidino-2-phenylindole (DAPI) (Abcam, Cambridge, UK) staining was performed. The samples were analyzed using a confocal laser scanning microscope.

### Statistical analysis

The results were presented as the mean ± standard deviation (SD). Statistical analysis was conducted using analysis of variance (ANOVA) followed by Tukey for multiple comparisons. Significance was defined as *p* < 0.05.

## Results

### Active ingredients of LTE

**Figure 2.**
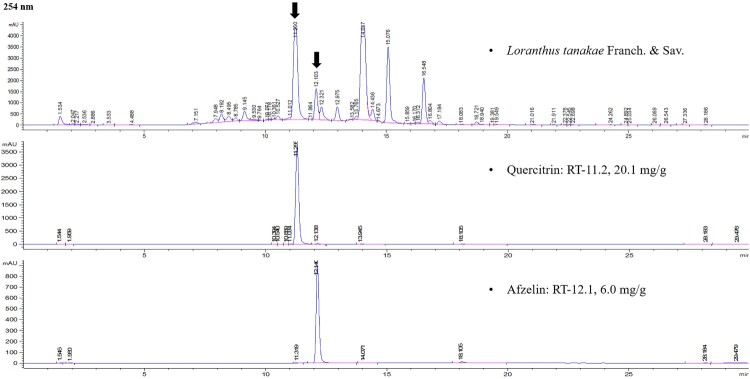
Analysis of LTE using HPLC-UV. Quercitrin and afzelin were detected. RT, retention time.

The LTE’s marker compounds were analyzed using HPLC-UV (Figure 2) and validated by comparing the retention time and consistency of the peaks to that of the standard compounds. The quercitrin and afzelin retention times were about 11.2 and 12.1 min, respectively, and their contents in LTE were approximately 1.98%−2.01% (19.8−20.1 mg/g) and 0.60%−0.63% (6.0−6.3 mg/g) at 254 nm.

### Effect of LTE on the production of inflammatory mediators in TNF-*α*/IFN-*γ*-stimulated HaCaT cells

**Figure 3.**
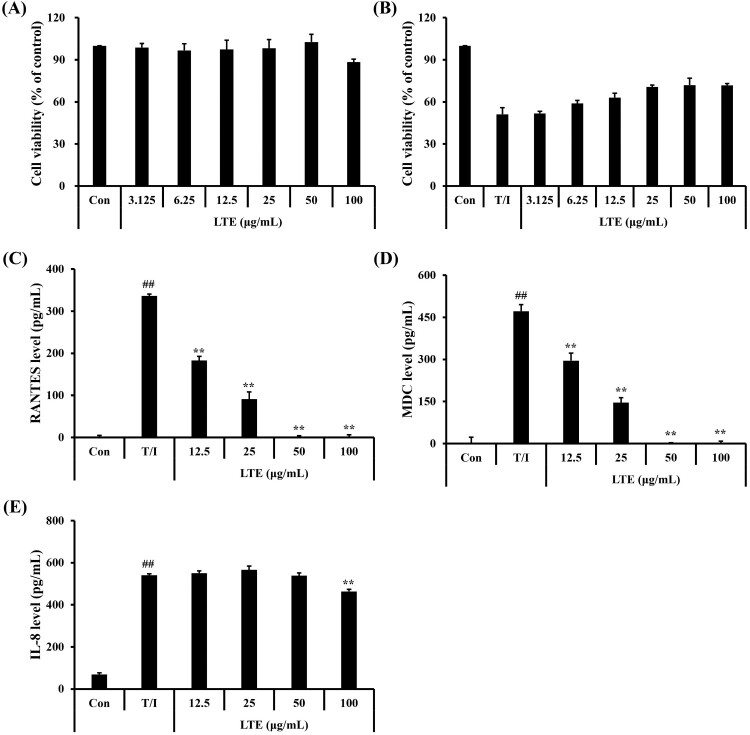
Effect of LTE on the production of inflammatory cytokines in TNF-*α*/INF-*γ*-stimulated HaCaT cells. (A and B) Cell viability (C) RANTES cytokine level, (D) MDC level, (E) IL-8 level. Data are the mean ± SD (*n* = 3). ^##^, vs. control, *p* < 0.01, **, vs. TNF-*α*/IFN-*γ*-stimulated cells, *p* < 0.01.

Cell viability tests showed that LTE at up to 100 μg/mL was not cytotoxic and was effective against TNF-*α*/IFN-*γ*-induced cytotoxicity (Figure 3(A and B)). The TNF-*α*/IFN-*γ*-stimulated HaCaT cells showed markedly increased levels of RANTES, MDC, and IL-8 compared with the non-treated cells (Figure 3(B–D)). LTE treatment significantly reduced the levels of RANTES and MDC in the TNF-*α*/IFN-*γ*-stimulated HaCaT cells in a dose-dependent manner, while IL-8 levels were significantly reduced only at the highest LTE concentration.

### Effect of LTE on P-JAK2 and P-STAT1 expression in TNF-*α*/IFN-*γ*-stimulated HaCaT cells

**Figure 4.**
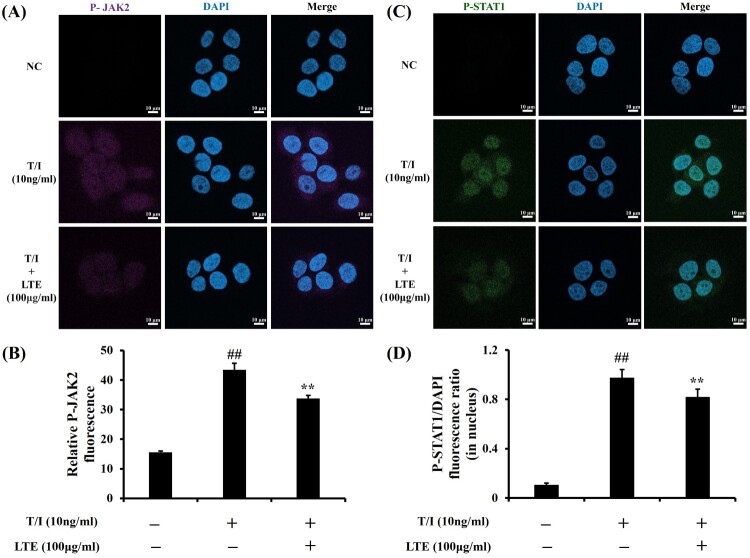
Effect of LTE on P-JAK2 and P-STAT1 expression in TNF-*α*/IFN-*γ*-stimulated HaCaT cells. (A) Representative figure for P-JAK2 expression. (B) Quantitative analysis of P-JAK2 expression. (C) Representative figure for P-STAT1 expression. (D) Quantitative analysis of P-STAT1. Scale bar = 50μm. Data are the mean ± SD (*n* = 3). ^##^, vs. control, *p* < 0.01, **, vs. TNF-*α*/IFN-*γ*-stimulated cells, *p* < 0.01.

The TNF-*α*/IFN-*γ*-stimulated HaCaT cells exhibited increased P-JAK2 expression compared with the non-treated controls (Figure 4(A and B)). The TNF-*α*/IFN-*γ*-stimulated cells’ treatment with LTE significantly reduced their P-JAK2 expression in comparison with the untreated cells. In addition, the LTE-treated TNF-*α*/IFN-*γ*-stimulated cells showed a marked increase in the translocation of P-STAT1 into the nucleus compared with the non-treated controls (Figure 4(C and D)). In contrast, the LTE treatment of the TNF-*α*/IFN-*γ*-stimulated cells significantly reduced the translocation of P-STAT1 into the nucleus compared with the untreated cells.

### Effect of LTE treatment on serum cytokine levels in OVA-induced AD mice

**Figure 5.**
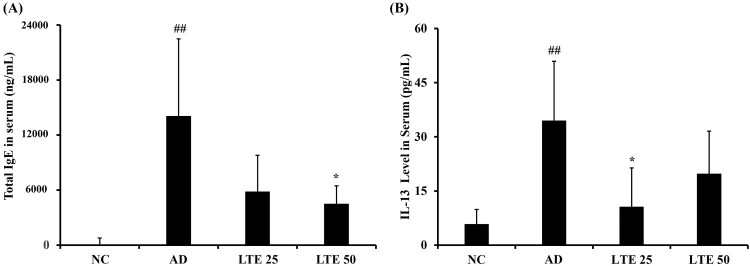
Effect of LTE on the levels of serum total IgE and IL-13 in OVA-induced AD mice**.** (A) Serum total IgE levels. (B) Serum IL-13 levels. Data are the mean ± SD (*n *= 6). ^##^, vs. NC, *p* < 0.01, *, vs. AD, *p* < 0.05.

The OVA-induced AD mice demonstrated a noticeable increase in serum total IgE levels compared with the control mice (Figure 5(A)); however, administration of LTE significantly reduced the serum total IgE compared with the AD mice. Similar to the results for serum total IgE, administration of LTE decreased the IL-13 serum levels (Figure 5(B)). In particular, treatment of OVA-induced AD mice with 50 mg/kg of LTE resulted in a significant difference in the serum IL-13 levels compared with that of the untreated AD mice.

### Effect of LTE treatment on skin inflammation in OVA-induced AD mice

**Figure 6.**
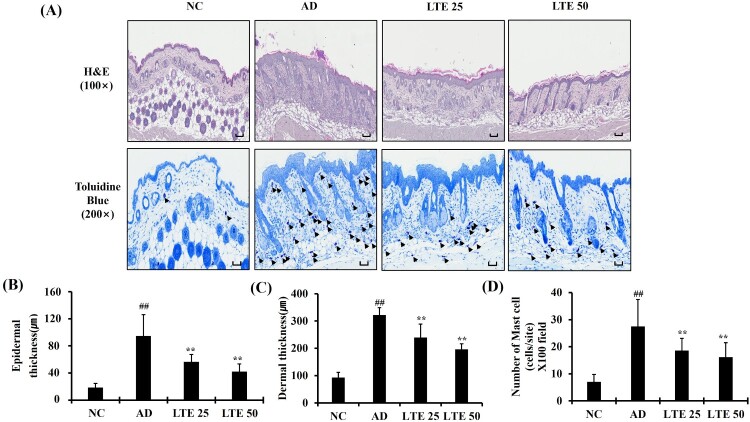
Effect of LTE on pathophysiological alteration of skin tissue in OVA-induced AD mice. (A) Representative figure for H&E and Toluidine blue staining. (B) Epidermal thickness. (C) Dermal thickness. (D) Number of mast cells. Scale bar = 50μm. Data are the mean ± SD (*n *= 3). ^##^, vs. NC, *p* < 0.01, **, vs. AD, *p* < 0.01.

The OVA-induced AD mice demonstrated an increased infiltration of inflammatory cells into damaged lesions compared with those of the normal control mice; this was accompanied by significant increases in epidermal and dermal thickness (Figure 6(A–C)). However, the OVA-induced AD mice administration of LTE demonstrated a significantly decreased infiltration of inflammatory cells compared with the untreated OVA-induced AD mic; this was accompanied by a reduction in epidermal and dermal thickness. In addition, OVA-induced AD mice showed a decidedly increased infiltration of mast cells compared with the normal control mice (Figure 6(A and D)). In contrast, treatment of OVA-induced AD with LTE significantly reduced the infiltration of mast cells compared with untreated OVA-induced AD mice.

### Effect of LTE on expression of P-JAK2 and P-STAT1 in OVA-induced AD mouse

**Figure 7.**
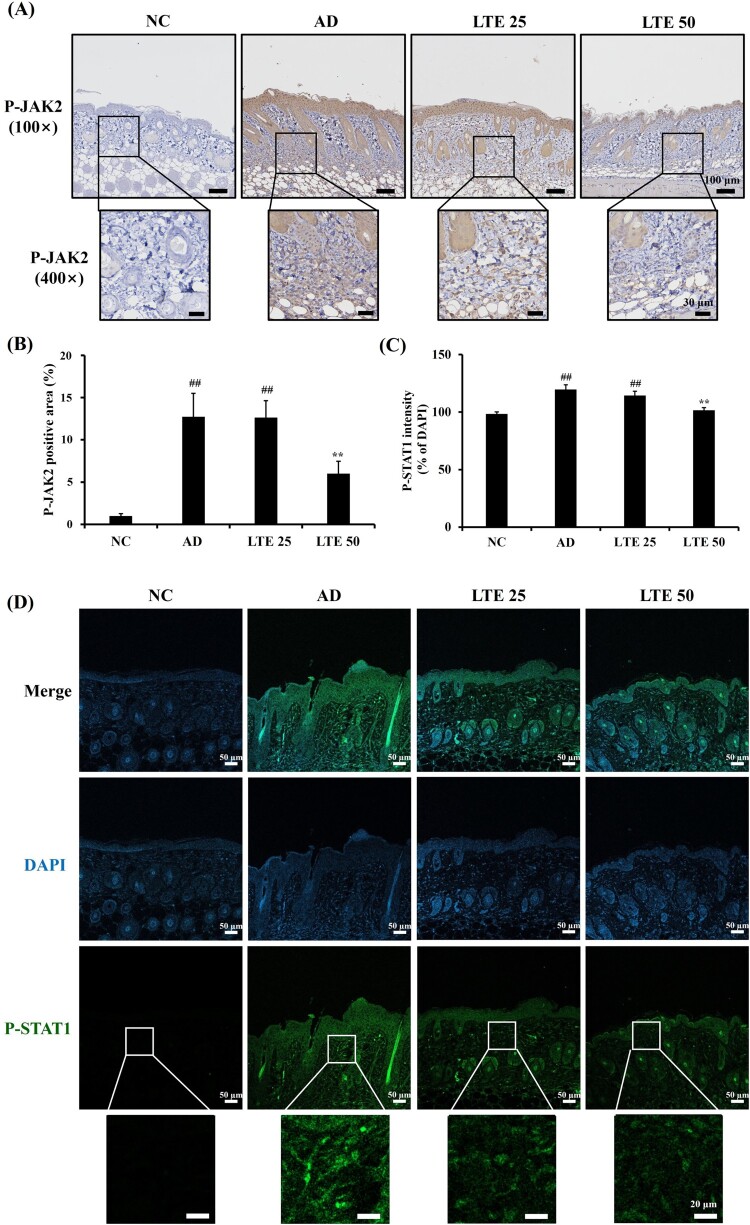
LTE downregulates the expression of P-JAK2 and P-STAT1 in OVA-induced AD mice. (A) Representative figure of P-JAK2 immunohistochemistry staining. (B) Quantitative analysis of P-JAK2 expression. (C) Quantitative analysis of P-STAT1 expression. (D) Representative figure of P-STAT1 immunofluorescence. Data are the mean ± SD (*n* = 3). ^##^, vs. NC, *p* < 0.01, **, vs. AD, *p* < 0.01.

The OVA-induced AD mice presented markedly increased P-JAK2 expression compared with the control mice (Figure 7(A and B)). After the administration of the LTE, there was a significant reduction in P-JAK2 expression compared with the untreated OVA-induced AD mice. In addition, the OVA-induced AD mice had markedly elevated P-STAT1 expression in comparison with the control mice (Figure 7(C and D)). However, the administration of LTE significantly decreased the P-STAT1 expression compared with the OVA-induced AD mouse.

## Discussion

AD is a chronic dermatological disease marked by severe itching and eczematous skin lesions, which are associated with abnormalities in the skin's barrier function, epidermal thickening, and IgE-mediated hypersensitivity to various antigens (Weidinger and Novak 2016; Yang et al. 2018). Currently, various therapeutics are used to treat AD; however, their use is limited because of their adverse effects and insufficient efficacy (Atherton 2003). Therefore, the development of therapeutics with fewer adverse effects and higher efficacy is required. *L. tanakae* is indigenous to South Korea and research has shown it to have many pharmacological properties. In particular, studies have reported that LTE's main components, quercitrin and afzelin, have anti-inflammatory effects and are effective against a variety of conditions, including respiratory, neurological, and skin diseases (Shin et al. 2013; Yin et al. 2013; Zhou and Nie 2015; Wang et al. 2022). In this study, we investigated the therapeutic effect of LTE on AD using TNF-*α*/IFN-*γ*-stimulated HaCaT cells and OVA-induced AD mice. Treatment using LTE significantly decreased the inflammatory mediators in TNF-*α*/IFN-*γ*-stimulated HaCaT cells, which was accompanied by decreased P-JAK2 expression and translocation of P-STAT1 into the nucleus. In addition, the administration of LTE significantly decreased the serum total IgE and IL-13 in OVA-induced AD mice, which was accompanied by reduced infiltration of inflammatory cells, particularly, mast cells. Consistent with the results of the *in vitro* experiment, the administration of LTE significantly reduced the expression of P-JAK2 and P-STAT1 in OVA-induced AD mice. These results from our experiments and other studies suggest that LTE has potential as a treatment for AD.

In the progression of AD, skin inflammation is associated with various cell types, of which keratinocytes are important in the development of skin inflammation (Yang et al. 2018). Keratinocytes release inflammatory mediators, such as cytokines and chemokines (Maeda and Yanagihara 2001). In particular, RANTES, MDC, and IL-8 are known to initiate skin inflammation (Tsuda et al. 2002). These mediators cause the infiltration of inflammatory cells into damaged lesions and induce Th2 differentiation, resulting in allergic responses. In this study, LTE treatment significantly reduced the inflammatory mediators in TNF-*α*/IFN-*γ*-stimulated HaCaT cells *in vitro* experiment. Additionally, the administration of LTE significantly decreased the infiltration of inflammatory cells, particularly mast cells, into damaged skin lesions. In addition, administration of LTE markedly reduced Th2 responses, including the elevation of serum total IgE and IL-13 in OVA-induced AD mice. Together, these results suggest that LTE treatment effectively suppresses skin inflammation in AD mice.

The JAK-STAT signaling pathway is involved in the development of AD skin lesions (Jin et al. 2009). Furthermore, the egg white protein OVA induces AD in mouse models, which is characterized by the infiltration of inflammatory cells and elevated levels of Th2 cytokines, such as IL-13 (Sung and Kim 2013). These mediators activate the JAK-STAT signaling pathway, which then induces Th2-driven immune responses, resulting in the promotion of B lymphocytes maturation related to excessive IgE production (Jeong et al. 2015). The binding of IgE to the skin’s mast cells triggers the release of histamine, which aggravates the AD condition (Bao et al. 2013). Additionally, the Th2-dominant environment prompts epidermal cells to produce various chemokines, pro-inflammatory cytokines, and angiogenic factors (Jeong et al. 2015). This leads to epidermal thickening, infiltration of inflammatory cells, and elevated expression of Th2 cytokines, which are crucial factors in the development of AD (Napolitano et al. 2023). In this study, LTE treatment was found to reduce the phosphorylation levels of JAK2 and STAT1 in both in vivo and in vitro experiments using the AD model. These changes in signal transduction activation led to reduced AD-associated skin inflammation. Therefore, this study’s findings suggest that the therapeutic effects of LTE on AD are associated with the suppression of the JAK-STAT signaling pathway induced by decreased IL-13 production.

In conclusion, this study’s results demonstrate that LTE exhibits potent anti-inflammatory and immunomodulatory effects in AD experimental models. In addition, the administration of LTE effectively inhibits the elevation of serum total IgE levels and pro-inflammatory cytokine production in AD experimental models; these effects may be attributable to the inhibition of JAK-STAT signaling mediated by decreased IL-13 levels. Therefore, we suggest that LTE is a potent therapeutic material for the treatment of AD.

## Data Availability

The data will be made available on request.
